# The long-term neurodevelopmental outcomes of febrile seizures and underlying mechanisms

**DOI:** 10.3389/fcell.2023.1186050

**Published:** 2023-05-25

**Authors:** You Yi, Chen Zhong, Hu Wei-wei

**Affiliations:** ^1^ Department of Pharmacology and Department of Pharmacy of the Second Affiliated Hospital, Key Laboratory of Medical Neurobiology of the Ministry of Health of China, Zhejiang University School of Medicine, Hangzhou, China; ^2^ Key Laboratory of Neuropharmacology and Translational Medicine of Zhejiang Province, Zhejiang Chinese Medical University, Hangzhou, China

**Keywords:** febrile seizures, disease occurrence, neurodevelopment, hippocampus, cortex

## Abstract

Febrile seizures (FSs) are convulsions caused by a sudden increase in body temperature during a fever. FSs are one of the commonest presentations in young children, occurring in up to 4% of children between the ages of about 6 months and 5 years old. FSs not only endanger children’s health, cause panic and anxiety to families, but also have many adverse consequences. Both clinical and animal studies show that FSs have detrimental effects on neurodevelopment, that cause attention deficit hyperactivity disorder (ADHD), increased susceptibility to epilepsy, hippocampal sclerosis and cognitive decline during adulthood. However, the mechanisms of FSs in developmental abnormalities and disease occurrence during adulthood have not been determined. This article provides an overview of the association of FSs with neurodevelopmental outcomes, outlining both the underlying mechanisms and the possible appropriate clinical biomarkers, from histological changes to cellular molecular mechanisms. The hippocampus is the brain region most significantly altered after FSs, but the motor cortex and subcortical white matter may also be involved in the development disorders induced by FSs. The occurrence of multiple diseases after FSs may share common mechanisms, and the long-term role of inflammation and γ-aminobutyric acid (GABA) system are currently well studied.

## 1 Introduction of febrile seizures

### 1.1 Definition and symptoms of febrile seizures

Febrile seizures (FSs) refer to convulsions induced by a sudden increase in body temperature (>38°C) in the absence of other underlying causes or disorders that induce convulsions, such as central nervous system (CNS) infections, electrolyte abnormalities, withdrawal, trauma, genetic predisposition or known epilepsy (Xixis et al., 2022). Children between the ages of about 6 months and 5 years old are the most likely to experience FSs. FSs are extremely common, occurring in up to 4% of children in this age group. Some children have a single FS event, and others have multiple events over early childhood. Approximately 30–40 percent of children who experience one FS will have a recurrence (Patel et al., 2015; Smith et al., 2019).

According to National Institutes of Health, symptoms of FSs are described as following: lose consciousness, both arms and legs will shake uncontrollably, eye rolling and rigid limbs. Sometimes during a FS, a child may lose consciousness but will not noticeably shake or move. FSs are categorized as either simple FSs or complex FSs: 1) simple FSs last no more than 15 min and this type does not recur within a 24-h period; 2) complex FSs last longer than 15 min, occur more than once within 24 h.

In recent years, the pandemic caused by 2019 coronavirus disease (COVID-19) has aroused widespread concern. One of the commonest clinical manifestations of COVID-19 is fever, and COVID-19 has become a common cause of FSs. As of April 2022, 13 million cases of COVID-19 have been reported among children and adolescents in the United States, accounting for nearly 20% of all cases in the United States. Among 15,137 children hospitalized for COVID-19, the most common neurological complication was FSs (3.9%) (Antoon et al., 2022). Although the incidence of seizures in children with fever during the COVID-19 pandemic has significantly increased (Iijima et al., 2022; Joung et al., 2023), in the latest retrospective case-control study, the risk of FSs secondary to COVID-19 did not increase compared to other causes (Hanlon et al., 2023). Meanwhile, a retrospective observational cohort study showed an increased frequency of complex FSs diagnoses during the COVID-19 pandemic (Cadet et al., 2023). Further analysis of the clinical features of FSs in patients with and without COVID-19 revealed that patients with FSs in COVID-19 tended to be predominantly male and had a later age of onset compared with non-Covid-19 patients (Seo et al., 2023). Because of the atypical age of onset and the greater likelihood of multiple convulsive episodes, patients (especially males) with COVID-19 require vigilance for FSs.

### 1.2 Therapeutics of FSs

Since the 1990 s, hundreds of articles have been published on the drug management of FSs. However, this has been a controversial area, and there are various views on drug management. This controversy reflects, in part, the fact that it is uncertain whether prophylactic medications with antiepileptics and antipyretics are effective without important adverse effects. There is no specific treatment for simple or complex FSs other than appropriate management of underlying etiologies driving the persistent febrile illness. Antipyretic drugs do not reduce the recurrence of FSs in children with a history of FSs (Lux, 2010). Diazepam in combination with acetaminophen reduced more seizure recurrences without serious adverse events compared with acetaminophen alone (Tanaka et al., 2022). In most patients in whom a febrile illness leads to frequent recurrence of FSs, studies have evaluated the use of benzodiazepines as a bridging measure during a subsequent febrile episode for several days (Printz et al., 2016; Guedj et al., 2017; Renda et al., 2020). Febrile status epilepticus can occur in less than 10% of children during the first FS. Rectal diazepam is used to abort this disorder if it lasts more than 5 min. There are also recommendations for intranasal midazolam. However, such treatment may have adverse effects on the child’s behavior and cognitive development (Xixis et al., 2022). The decision to treat thus requires assessment of the potential risks and benefits to children. Phenobarbital at times of fever has been proven to be ineffective, probably because of the delay in achieving appropriate serum and tissue levels (Farwell 1990; Herranz 1988). To avoid the side effects of continuous antiepileptic drugs (AEDs), rapid-acting antiepileptics given only during fever periods have been used in an attempt to reduce the risk of recurrent FSs. To date, only prophylactic diazepam (administered orally or rectally) has been studied in placebo-controlled trials. Intermittent diazepam reduces the recurrence rate in children with FSs, but the incidence of adverse effects is as high as 30% (Offringa et al., 2017; Offringa et al., 2021). Current reports have confirmed the exact efficacy of levetiracetam with an extremely low incidence of adverse effects. If this effect is repeated in other studies, levetiracetam may be considered as a prophylactic treatment option in selected families where anxiety over potential FSs recurrence is high. Melatonin treatment had a similarly low incidence of adverse events, and further studies are needed to demonstrate its effectiveness *versus* placebo (Xixis et al., 2022).

## 2 Etiology of FSs

### 2.1 Generation of FSs

Any fever of adequate height may cause FSs. Some clinical studies found that upper respiratory tract infection was determined the commonest disease (81.6%) in FSs followed by acute gastroenteritis (15.4%) and urinary tract infection (3%), respectively (Gunes et al., 2021), but no specific febrile cause was more likely to cause FSs. A large number of clinical and preclinical studies have uncovered the pathological features and possible etiological factors of FSs, such as mutation of fever response genes and γ-aminobutyric acid (GABA) gene, ion channel activity and inflammation (Hessel et al., 2014; Chen et al., 2019). However, there is no generally acknowledged mechanism of FSs.

Briefly, mutation in *SCN1A* gene, encoding the voltage-gated sodium channel (VGSC) NaV1.1, is widely recognized as a leading cause of genetic FSs, due to the decrease in the Na^+^ current density, mainly affecting the inhibitory neuronal transmission (Kasperaviciute et al., 2013; Scalise et al., 2020; Scalise et al., 2022). However, failure to detect association between polymorphisms of *SCN1A* and FSs in Chinese patients suggests that the correlation between *SCN1A* mutation and FSs may just consist in specific races (Zhang et al., 2010). Mutation in Na^+^ -channel beta1 subunit gene *SCN1B*, which usually leads to loss of function, is also associated with FSs (Wallace et al., 1998; Audenaert et al., 2003; Steinlein, 2004). *SCN2A* is a well-established epilepsy gene, encoding the neuronal sodium channel NaV1.2, may also contribute to the generation of FSs (Saitoh et al., 2015; Berseem et al., 2022; Skotte et al., 2022). *GABRG2* mutation associated with FSs has been elucidated (Audenaert et al., 2006; Todd et al., 2014; Haerian et al., 2016). Some other gene mutations are also identified, such as *SRP9*, *ADGRV1* and the fever response genes *PTGER3*, *IL-6* and *IL-10* (Shahrokhi et al., 2017; Chen et al., 2019). Last year, the largest genetic study of FSs to date further identified several novel loci strongly associated with FSs. Variants at two loci were functionally associated with altered expression of the fever responsive genes *PTGER10* and *IL-2*, and the other four loci contained genes (*BSN*, *ERC2*, *GABRG1*, *HERC1*) influencing neuronal excitability by regulating neurotransmitter release and binding, vesicular transport or membrane trafficking at synapses (Hessel et al., 2014; Han et al., 2020; Skotte et al., 2022). Mutations in several genes strongly associated with fever related epilepsy syndromes are also notable, such as *STX1B*, encoding a presynaptic protein, and *CELF4* haploinsufficiency mutations, encoding a splicing regulator (Halgren et al., 2012; Schubert et al., 2014). FSs are serious adverse events following measles, mumps and rubella (MMR) vaccination. A genome-wide association scan identified two loci clearly associated with MMR related febrile seizures, harboring the interferon stimulated gene *IFI44L* and the measles virus receptor *CD46*. Two loci associated with FSs in general, *ANO3* (also known as *TMEM16C*), and locus associated with serum magnesium levels have also been identified (Feenstra et al., 2014; Sisodiya, 2014).

Similar to epilepsy, the onset of FSs is also closely associated with neural excitation-inhibition imbalance. Ion channels are key gating channels that control intra and intercellular ion currents as the important regulators of neuronal network excitability. The direct effect of heat on ion channels localized to the site of action potential initiation potentially causes a profound increase in neuronal excitability, which is likely to contribute to FSs genesis (Thomas et al., 2009). At present, several ion channels are known to be involved in FSs, including KCC2 (K^+^ -Cl^−^ co-transporter, the chloride exporter), NKCC1(Na^+^-K^+^-2Cl^−^ cotransporter, the chloride importer) (Asisipo et al., 2020), TRPV1 (transient receptor potential vanilloid-1) (Barrett et al., 2018) and TRPC3 (a member of canonical transient receptor potential channel) (Sun et al., 2018).

Inflammatory elements have major roles in FSs pathogenesis. A meta-analysis showed that IL-6 (−572, −174, −597) polymorphisms were significantly associated with susceptibility to FSs (Chen et al., 2019). At the protein level, several meta-analyses and researches on animal models suggest that cerebrospinal fluid (CSF) cappase-1/IL-1β level, and serum high mobility group box protein 1 (HMGB1), tumor necrosis factor-α (TNF-α), IL-1β, IL-6 level are associated with an increased risk of FSs in children (Choi et al., 2011; Kwon et al., 2018; Tang et al., 2020; Carman et al., 2021). These inflammatory cytokines impact FSs through changing neuronal excitability, regulating GABAergic transmission, inhibiting BDNF-TrkB signaling or enhancing the NF-κB pathway (Wang et al., 2019; Sun et al., 2020; Tang et al., 2020).

There are other hypotheses about FSs, for instance, respiratory alkalosis induced by hyperthermia (Schuchmann et al., 2006; Barrett et al., 2018), iron deficiency (Vaswani et al., 2010; Papageorgiou et al., 2015), and the role of immunoreactive-arginine vasopressin and immunoreactive-somatostatin (Nagaki et al., 1996) (Supplementary Table S1).

### 2.2 Recurrence of FSs

According to some follow-up studies, the risk of FSs recurrence decreased linearly with increasing age (−2% per month) and the risk was higher among patients with abnormal electroencephalogram (EEG) (Cappellari et al., 2018). Younger age at first seizure, short duration of fever before the onset of first FS, lower temperature at onset, and family history of FS are risk factors of recurrence of FSs in children (Patel et al., 2015; Kumar et al., 2019).

Currently, however, the recurrence of FSs cannot be effectively predicted, and reliable biomarkers and diagnostic evidence are lacking. Current researches on the mechanisms of recurrence are still progressing. A case-control study in a Romanian pediatric population reported that recurrent crises and repeated episodes of seizures are more frequent in the *GABRG2* Asn196Asn TT genotype polymorphism, with 8 times higher risk of developing recurrent FSs (Butila et al., 2018). TRPV1 is a nonselective cation channel, as a key component implicated several inflammatory diseases. A massive amount of evidence has demonstrated that TRPV1 is extensively expressed in the CNS and there might be a close relationship between TRPV1 and neuroinflammation (Kong et al., 2014; Yang et al., 2019; Zhang et al., 2021). It is found that TRPV1 promotes recurrent FSs by increasing pro-inflammatory cytokines (Huang et al., 2015), and TRPV1 was mainly derived from microglia to participate in neuroinflammatory response and recurrent FSs (Kong et al., 2019). There are also several blood analyses. C-reactive protein level, blood glucose level, serum sodium level, serum zinc level and vitamin D were indicated significant association with recurrent FSs (Hugen et al., 1995; Kiviranta and Airaksinen, 1995; Lee and Kim, 2012; Waqar Rabbani et al., 2013; Arul et al., 2020; Bhat et al., 2020; Kubota et al., 2021; Miyagi et al., 2022). To be noted, there is evidence from meta-analysis and cross-sectional studies that hyponatremia and hypozinc may be strongly associated with the recurrence of FSs. However, there is a lack of direct evidence to show whether sodium or zinc supplementation can effectively prevent or treat recurrence of FSs, and it is also unclear whether monitoring blood sodium or zinc in children after first FS is effective in predicting recurrence of FSs. At present, only randomized clinical trials with few sample sizes have found that zinc supplementation can effectively reduce the recurrence rate of FSs (Fallah et al., 2015). The association of hyponatremia with FSs recurrence may be related to mutations in sodium channels in the etiology of FSs, but the evidence is weak (Supplementary Table S2).

## 3 The long-term adverse outcomes associated with FSs

Electroencephalographic and biochemical long-lasting abnormalities have been reported both in clinical cases and animal models of FSs (Leaffer et al., 2013; Mohammed et al., 2017). At the outset, scholars focused on the association between infant FSs and epileptogenesis in adulthood. More and more data suggest that FSs may also contribute to damage of cognition, motor skills and mental diseases (Leaffer et al., 2013; Rajab et al., 2014; Crespo et al., 2018; Lin et al., 2021; Harris et al., 2022).

### 3.1 Diseases or pathological manifestations after FSs

#### 3.1.1 Epileptogenesis

In 1997, a study of 44 adult patients suggests that a history of FSs is associated with the subsequent temporal lobe pathology (Barr et al., 1997). The association between temporal lobe epilepsy (TLE) and the history of FSs has since gradually come into public view. There have been numerous cohort studies designed one after another to investigate the association of FSs with epilepsy in adult, with numbers of patients involved ranging from hundreds to millions and time span more than 10–20 years. Although the proportions varied from 5 to 18 folds, these data suggest a significant increase in the incidence of epilepsy after FSs (Vestergaard et al., 2007; Neligan et al., 2012; Chiang et al., 2018; Tsai et al., 2018). Further, of those individuals who experienced FSs, the frequency of subsequent development of epilepsy was 2.15-fold greater in females, 4.846-fold greater in patients with recurrent FSs (Chiang et al., 2018). Researchers assessed predictive value of epileptiform discharges for subsequent epilepsy after FSs and found that patients with normal EEGs were unlikely to develop epilepsy (Kavcic and Rener-Primec, 2018). The important impact of FSs on subsequent epileptogenesis has been recognized, but over a large time span, it is clinically difficult to validate directly. Therefore, animal models of FSs are well-established and the research achievements are considerable.

Although there are some data showing that prolonged experimental FSs lead to adult-onset TLE (Dube et al., 2006), most data suggest that a single episode of prolonged FSs may not induce *per se*, but accelerates epileptogenesis, and increases seizure susceptibility and severity (Dai et al., 2014; Hamelin et al., 2014; Dutton et al., 2017; Alese et al., 2020). Notably, in animal models, FSs were found to cause interictal epileptifom EEG abnormalities, which is consistent with clinical studies, suggesting detection of EEG may serve as an important means of predicting epileptogenesis after FSs. These data were obtained in adulthood, and whether FS also has alterations in EEG during development requires more evidence. Current studies only found that the seizure susceptibility decreased in 35-day-old (P35) FS rats but increased in P60 FS rats (Feng et al., 2015), but the EEG changes during neurodevelopment until adult were not examined. Dai’s findings provide direct evidence of sex-dependent acquired seizure susceptibility after complex FSs (Dai et al., 2014). Female FSs rats were more susceptible to pentylenetetrazol and maximum electric shock than male FSs rats. The protein expression of interleukin-1β (IL-1β), an inflammatory factor associated with seizure susceptibility, was higher in adult FSs females than in males, which may reflect a gender-specific phenomenon of seizure susceptibility.

#### 3.1.2 Cognitive and memory impairments

Recognition memory is impaired in children after FSs and this memory impairment can persist for at least 10 days to 1 year (Martinos et al., 2012). Cognition impairment, including perceptual reasoning and working memory defects, were identified in patients aged 6–12 years with FSs onset at the age of 2–2.5 years old (Tsai et al., 2015). A generation R study suggested that children with recurrent FSs might be at risk for delayed language development around 6 months after FSs (Visser et al., 2012). Similarly, 4-year-old children with FSs had been identified at child healthcare centers in Gothenburg. One-third of the children had at least one neurodevelopmental disorder diagnosis or marked developmental problems within areas of attention, speech and language or general cognition. No differences were found between children with single vs. recurrent FS or simple vs. complex FSs (Nilsson et al., 2019). These clinical studies did not analyze the key brain regions or molecular targets affected. In addition, reports on whether these cognitive and language impairments extend into adulthood are lacking.

In experimental FSs model, most agree that FSs cause moderate memory impairment, which focus on spatial, working and reference memory (Chang et al., 2003; Dube et al., 2009; Kloc et al., 2022). FSs had transient effects on spatial learning in immature rats in a few days (Yagoubi et al., 2015). But spatial memory problems were identified in male adult rats following experimental prolonged FSs. Remarkably, possible mechanisms underlying these deficits may involve hippocampal impairments of dendritic filtering of cortical inputs discoordination of entorhinal-hippocampal circuit, as well as neuron restrictive silencing factor mediated aberrant generation of excitatory synapses in the dentate gyrus, that results in dendritic loss in the hippocampus (Rajab et al., 2014; Patterson et al., 2017; Kloc et al., 2023). It was interesting that sex seemingly had a remarkable effect on spatial cognitive outcome where adult males with FSs fared worse than adult females with FSs (Kloc et al., 2022). At the same time, there are some reports that FSs also resulted in adult memory deficits in novel object recognition task, inhibitory avoidance task, and contextual fear conditioning task and inhibition of TRPV1 receptors during FSs in part prevented learning deficits in adult (Dai et al., 2019; Harris et al., 2022). Interestingly, prolonged FSs in infant rats caused adult memory deficits that could be transmitted to the next-generation, mainly through the mother, and may be associated with DNA methyltransferase (DNMT) 1 upregulation (Dai et al., 2019). These animal findings pinpoint that FSs may cause cognitive impairment that persists into adulthood. Mechanisms of sex differences and inheritance to the next-generation are insufficiently studied and will be research directions with great clinical significance.

#### 3.1.3 Psychiatric disorders

It was showed the most common psychiatric diagnoses after FSs were anxiety, attention-deficit/hyperactivity, and personality disorders (Dreier et al., 2020). The cohort studies that found the association between FSs and epilepsy also identified a 11.26-fold higher frequency of comorbid autism after FSs compared with controls (Chiang et al., 2018). To exclude the disturbances of epilepsy and focused on analyzing the contribution of FSs *per se* to psychiatric disorders, one evaluation of long-term risk of psychiatric disorders among 2103232 children with recurrent FSs from Denmark at Aarhus University was conducted. A history of recurrent FSs appears to be associated with a risk of psychiatric disorders, including organic mental disorders, affective mental disorders, schizophrenia, mood disorders, somatoform disorders, intellectual disability, disorders of psychological development, etc. (Dreier et al., 2019). In animal studies, the results of depression-like behavior in rats after FSs showed that FSs significantly reduced the sucrose consumption in the sucrose preference test and increased the immobility time in the forced swim test in P37 and P60. This study also demonstrated that FSs caused depression-like behavioral changes that may be inherited to the next-generation of rats and leaded to increased mGluR3 mRNA expression and mGluR1 gene hypermethylation in two generations of rats (Alese and Mabandla, 2019). But there is no direct evidence that mGluR3 and mGluR1 regulate the inheritance of this depressive phenotype.

#### 3.1.4 Attention deficit hyperactivity disorder (ADHD)

After comparison of relation between ADHD in children with and without FSs, it was concluded that hyperactivity has a significant relation with FSs in male gender (Salehi et al., 2016). After 11 years of follow-up and identified 1,081 children with FSs as the case cohort, Ku et al. found that FSs may increase the risk of subsequent ADHD occurrence in children (Ku et al., 2014). It is reported that the overall risk of ADHD in the FSs+/preterm + group was higher than that in the FSs+/preterm-group. Preterm birth may be a risk factor for subsequent ADHD in children with FSs (Lin et al., 2021). A population-based cohort of 906,379 children born in Denmark were followed up for 22 years. The findings indicated an association between FSs and subsequent development of ADHD, after adjusting for socioeconomic and perinatal risk factors, and family history of epilepsy, or psychiatric disorders (Bertelsen et al., 2016).

#### 3.1.5 Mortality

In current research, the presence or absence of a history of FS episodes had little effect on long-term mortality. 132 of 100 000 children died within 2 years of FSs compared with 67 deaths per 100 000 children without a history of this disorder (Vestergaard et al., 2008). In the nested case-control study, children with simple FSs had a mortality rate similar to that of the background population, whereas mortality was increased for those with complex FSs. This finding can be partially explained by pre-existing neurological abnormalities and subsequent epilepsy. In aforementioned study from Denmark at Aarhus University, increased mortality was found in individuals with a history of FSs who later developed epilepsy (Dreier et al., 2019). In summary, long-term mortality is not increased in children with FS, but there seems to be a small excess mortality after complex FS (Vestergaard et al., 2008). So, we should be reassured that death after FSs is not increased.

### 3.2 Long-term alterations on tissues or cells after FSs

#### 3.2.1 Temporal abnormalities: hippocampus and amygdala

At the time of the observation of increased epilepsy susceptibility, researchers found substantial hippocampal and amygdala changes after FSs. The abnormalities of hippocampus are the most clinically reported. There are some case reports, which found development of hippocampal sclerosis after FSs (Sokol et al., 2003; Merkenschlager et al., 2009). When magnetic resonance imaging (MRI) was done immediately (a few days after FS), it was showed that T2 weighted signal and the volume of hippocampus increased (swelling) (Sokol et al., 2003; Yokoi et al., 2019). In children with FSs from 3 to 23 months later, MRI imaging found a markedly hyperintense hippocampus (Provenzale et al., 2008). In their teens 2-3 years after FS onset, hippocampal subfield volumes were reduced (Grunewald et al., 2001; Finegersh et al., 2011; Peng et al., 2021), and these abnormalities were probably more pronounced in men (Auer et al., 2008). These suggest that FSs cause parenchymal lesions in the hippocampus, which may have undergone complex lesions during development. A more nuanced analysis noted an increase of hippocampal calretinin-immunoreactive neurons after FSs (Blumcke et al., 1999). Additional clues found that sulfur dioxide (SO_2_) content is overproduced during the development of FSs and related brain injury (Yang et al., 2018). Still, it remains to be elucidated, how these changes contribute to the pathogenesis of TLE and when is the key time nodes for intervention and treatment.

Preclinical studies have also found that FSs led to a temporal morphological disturbance. MRI histology showed increased fiber density and anisotropy in the hippocampus, and reduced neuronal surface area in the amygdala of rats. Diffusion tensor imaging (DTI) abnormalities were detected in the amygdala and persisted up to 8 weeks (Jansen et al., 2008). The cell numbers decreased by 10% in the CA1 and hilus but did not reduce in the CA3 or dentate gyrus areas, suggesting that the temporal abnormalities after FSs not only changed over time, but possibly also differed by brain regions. Meanwhile, functional impairments were more robust. Shortly after FSs, long-term potentiation (LTP) in CA3-CA1 synapses was strongly reduced (Postnikova et al., 2021). FSs reduced thresholds to chemical convulsants in the immature rat model and electrical stimulation *in vitro*, suggesting FSs enhanced hippocampal excitability long term during development (Dube et al., 2000). The modification of neuronal excitability of limbic circuits in the developing brain induced by FSs may last into adulthood (Chen et al., 1999). Thus, alterations in temporal lobe tissue morphology after FSs may stem from neuronal excitotoxicity that provoke more long-lasting signals or effects persisting into adulthood.

To identify molecular candidates, which entrain this structural and functional re-organization, Bart C Jongbloets et al. investigated temporal changes in mRNA expression profiles 1 h to 56 days after FSs. They screened 931 regulated genes and profiled several candidates using *in situ* hybridization and histology at 3 and 14 days after FSs. Temporal regulation of multiple processes was identified, such as stress-, immune- and inflammatory responses, glia activation, glutamate-glutamine cycle and myelination (Jongbloets et al., 2015), indicating the complex short- and long-term changes of hippocampus after FSs. More studies on specific molecules at specific developmental stages need to be further promoted to deconstruct the long-term effects of FS on the temporal lobe and subsequent epileptogenesis.

#### 3.2.2 Alterations of cortex

The effects on the hippocampus after FSs are probably the most profound and thoroughly studied (Nazem et al., 2012; Raijmakers et al., 2016), but the role of FSs on other brain regions should not be ignored, especially we note that FSs not only cause alterations of hippocampus related disease in adulthood, but also similarly affect the occurrence of disorders related to cortex, such as ADHD and other psychiatric disorders. Moreover, in the previously mentioned study in Gothenburg, FSs children had motor functioning impairment (Nilsson et al., 2019). Experiment FSs caused fine motor coordination impairment and gait disturbances (Crespo et al., 2018). These suggest that motor related motor cortex and other brain regions may have been affected by FSs.

The vast majority of hippocampal recorded seizures were preceded by a drop in cortical EEG amplitude that began half a minute before hippocampal spasms and was sustained after seizure termination (Dube et al., 2006). This suggests that the limbic spontaneous seizures exert a substantial disturbance to normal cortical neuronal activity. A significant increase in A1 receptor density and mRNA coding A1 was observed in cerebral cortical area 48 h after FSs. In contrast, a significant decrease in A2A receptor density and 5′-nucleotidase activity was detected 48 h after FSs (Leon-Navarro et al., 2015). These results illustrated that long-term alterations after FSs may also exist in the cortex. Whether these cellular molecular abnormalities detected in the cortex are maintained to a longer time and thus provide an explanation for functional abnormalities in adulthood also awaits more in-depth studies.

#### 3.2.3 White matter tract reorganization

MRI studies have demonstrated acute and long-term subcortical white matter changes following FSs (Takanashi et al., 2006; Pujar et al., 2017). Between 3 and 9 days after FSs, white matter lesions were observed and the diffusion abnormality disappeared between days 9 and 25 (Takanashi et al., 2006). In a homogeneous, population-based sample, fractional anisotropy in early maturing central white matter tracts and mean and axial diffusivity in several late-maturing peripheral white matter tracts were found to increase 8 years post-FSs (Pujar et al., 2017). Drug resistant TLE is increasingly recognized as a system level disorder affecting the structure and function of large-scale gray matter networks, while the superficial white matter, close to neocortical regions, plays a key role in maintaining cortical connectivity, which is associated with seizure outcome (Liu et al., 2016). Long lasting abnormalities of white matter after FSs therefore need to be paid more attention and may be one of the etiologies of drug-resistant TLE.

#### 3.2.4 Activation of microglia

In the identification of mRNA expression profiles following FSs described above, glia was activated (Jongbloets et al., 2015). Among these, modulation of microglia in the structure and function of the hippocampus after early-life FSs received much attention (Andoh et al., 2020; Weninger et al., 2021). Massive microgliosis after FSs was found (Weninger et al., 2021). The microglial expression levels of Iba1 and ED1 (lysosomal markers) and the proportion of microglia with amoeboid morphology increased, indicating that microglia were activated after FSs (Kong et al., 2019). The expression levels of microglial cytokines like inflammatory cytokines such as TNF-α and IL-1β were elevated 4 h after FSs (Kim et al., 2015). In addition, microglial synaptic displacement in motor cortex is a protective event during the pathological process of complex FSs. There is a significant increase of perisomatic GABAergic synapses around neuronal soma after complex FSs. So, complex FSs caused enhanced neuronal GABA transmission and increased neuronal excitability, as GABAergic synaptic transmission plays an excitatory role in juvenile stage. Microglia extensively associated with glutamatergic neuronal soma, displacing but not engulfing GABAergic synapses around neuronal soma to reduce the GABA transmission and neuronal excitability through P2Y_12_ receptors. These studies provide a rationale to elucidate the protective role of microglia and may be a potential future treatment for complex FSs (Wan et al., 2020). Microglial displacement of GABAergic synapses also provides neuroprotection in the adult brain (Chen et al., 2014). In addition, microglia can also secrete numerous neurotrophic factors to elaborate repair of injured neurons after FSs (Parkhurst et al., 2013; Araki et al., 2021). The induction of brain-derived neurotrophic factor (BDNF) mRNA was firstly observed in the dentate gyrus at 30 min after FSs, peaked at 3 h and returned to basal level at 24 h. It was also observed in the CA3 of hippocampus from 2 to 3 h (Kim et al., 2001). BDNF play critical role in neuronal survival, synaptic plasticity and cognitive functions and is known to mediate its action through various intracellular signaling pathways triggered by activation of tyrosine kinase receptor B (TrkB) (Pandya et al., 2013). These observation times did not last into adulthood, and the long-term role of microglia after FSs remains to be explored.

## 4 Possible mechanisms for long-term neurodevelopmental outcomes of FSs

**FIGURE 1:**
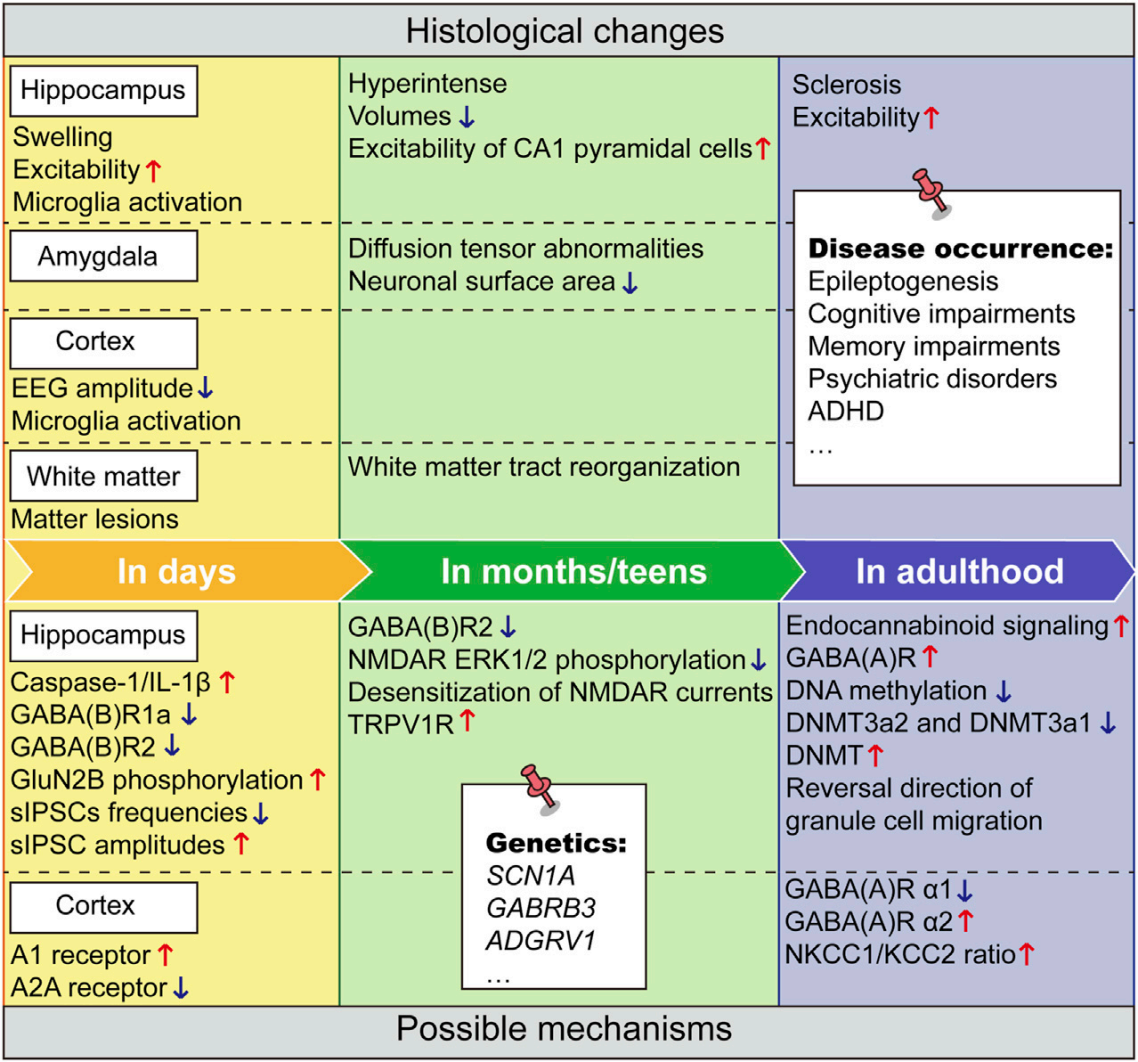
The neurodevelopmental changes after FSs and underlying mechanisms over time. The upper part summarizes the histological changes over time following FSs and the lower part summarizes the corresponding underlying mechanisms. Upward red arrows indicate increase or enhancement, and downward blue arrows indicate decrease, attenuation, or loss. The contents on the white notes indicate lesions or gene mutations that may span a long-time course throughout the developmental stage.

Clinically, it is somewhat difficult to find targets that dynamically change over prolonged periods after FSs. Most of the mechanistic studies are based on animal models of experimental FSs and often lack clinical proof of correspondence. But these studies remain valuable. Overall, the current mechanistic hypotheses for neuropsychiatric damage from FS are summarized (Figure 1).

### 4.1 Endocannabinoid system

Feng’s work identified prolonged increase of endocannabinoid signaling in adult seizure susceptibility following FSs, as a potential therapeutic target for preventing the development of epilepsy after infantile FSs (Feng et al., 2016). In a study of long-term plasticity following FSs induced endocannabinoid signaling, the activity dependent retrograde inhibition of endogenous cannabinoids on GABA release continued to increase in the hippocampus of rats (Chen et al., 2003). Endogenous cannabinoid system has been reported to be associated with depression, ADHD and other diseases. Targeting the endocannabinoid system has the potential to alleviate depression (Bright and Akirav, 2022). Clinical randomized experiments found reduced symptoms and cognitive impairment in some ADHD patients after cannabinoid use (Cooper et al., 2017). Recent evidence suggests that modulation of endocannabinoid tone has anxiolytic or antipsychotic effects (Navarrete et al., 2020). This undoubtedly suggests that endocannabinoid signaling may not only be involved in hippocampal related epilepsy and functional alterations after FSs, but may also play a key role in other disease courses.

### 4.2 Inflammation

In examination of the molecular changes in the rat brain after FSs throughout the latent period, the largest changes were for genes involved in inflammation signaling. Reduction of early inflammatory responses after FSs reduced the risk of subsequent spontaneous seizures (Jung et al., 2011). An important role for caspase-1/IL-1β inflammatory signaling pathway was identified in the increased susceptibility to seizures induced by FSs. Caspase-1/IL-1β expression was upregulated transiently at the onset of FSs. In neonatal mice, caspase-1 inhibitor CZL80 markedly reduced neuronal excitability and incidence of FSs, and, in adult mice, relieved later enhanced epileptogenic susceptibility (Tang et al., 2020). When IL-1β was given after FSs, the incidence of spontaneous seizures in rats will be significantly increased (Fukuda et al., 2015). Increased GluN2B phosphorylation at Tyr1472 site mediated by the transient increase of IL-1β was involved in the enhanced adult seizure susceptibility after FSs and showed a 3-day therapeutic time-window for reversing the enhanced seizure susceptibility after FSs (Chen et al., 2016). It is interesting that FSs or early-life IL-1β treatment increased the expression of cannabinoid type 1 receptor (CB1R) for over 50 days, which was blocked by IL-1Ra or was absent in IL-1R1 knockout mice (Tang et al., 2020). Knockdown and synthesis inhibitor of endocannabinoid abolished FSs or IL-1β-enhanced seizure susceptibility. So, the long-term effects of FSs on IL-1β and endocannabinoid may have crosstalk. Furthermore, IL-6 and IL-8 in the IL-1 cytokine system are closely associated with FSs. Compared to children without hippocampal signal abnormalities, children with T2 hippocampal hyperintensity on MRI after FSs had significantly higher IL-8 and IL-6 levels and lower IL-1Ra/IL-6 and IL-1Ra/IL-8 ratios. The lower IL-1Ra/IL-6 ratio was highly predictive of T21 hyperintensity in hippocampus after FSs (Gallentine et al., 2017; Vezzani et al., 2019), suggesting that IL-1Ra and IL-6 may also be powerful biomarkers and potential therapeutic targets for hippocampal injury after FSs.

### 4.3 GABAergic and glutamatergic system

Within the molecular examined in the rat brain after FSs throughout the latent period, the largest changes were the genes involved in inflammation signaling, then were (GABA signaling and glutamatergic system (Jung et al., 2011). We know that the neural network is overexcited during the onset of FSs, and this excitement may last for some time after the onset (Reid et al., 2013). Swijsen et al. evaluated BrdU-labeled Devil Gundam (DG) cells for co-expression with GABA A receptors [GABA (A)Rs] and N-methyl-D-aspartate receptors (NMDARs). The number of BrdU-GABA (A)R co-labeled cells not BrdU-NMDAR co-expressing cells was increased in adult after FSs. The results demonstrate that developmental seizures cause a long-term increase in GABA (A)R expression in newborn DG cells (Swijsen et al., 2012b). In other research, increased adult hippocampal protein expression of NR2B was found after early life inflammation and FSs. The inflammation + FSs group had also decreased protein expression of GluR2 and GABAA α1 receptor subunits and mRNA and protein expression of KCC2 (Reid et al., 2013). In the adult sensorimotor cortex, significantly lower levels of the GABA (A) receptor α1 subunit, higher levels of the α2 subunit, and a higher NKCC1/KCC2 ratio were reported in FSs rat (Reid et al., 2012), but the functional outcome and disease occurrence during adulthood due to these alterations remain to be investigated. Therefore, the GABAergic system may be more markedly altered after FSs, while the glutamatergic system may be more likely to change concomitantly in expression due to other factors such as inflammation and different brain regions. It is also found that experimental FSs induce changes in GABA (A) R-mediated neurotransmission in the dentate gyrus. Frequencies of spontaneous inhibitory postsynaptic currents (sIPSCs) were reduced in FS rats, whereas sIPSC amplitudes were enhanced (Swijsen et al., 2012a). GABA (B)R1a and GABA (B)R2 subunits and the binding of the 2 subunits decreased in hippocampus after FSs in immature rats. To be noted, the decrease of GABA (B)R1a lasted for 15 days but that of GABA (B)R2 persisted for more than 30 days. These changes may result in long-lasting imbalance of excitation/inhibition function in hippocampus (Han et al., 2006). Aberrant migration of neonatal-generated granule cells resulted in granule cell ectopia that persists into adulthood. FSs induced an upregulation of GABA (A) receptors [GABA (A)-Rs] in neonatally generated granule cells, and hyperactivation of excitatory GABA (A)-Rs caused a reversal in the direction of granule cell migration. This abnormal migration was prevented by RNAi-mediated knockdown of the NKCC1, which regulates the excitatory action of GABA (Koyama et al., 2012). These all may be the neuropathological basis for the subsequent FSs induced excitation/inhibition imbalance in adulthood and holds promise as potential intervention targets for disorders such as epilepsy.

Although NMDARs expression does not change obviously after FSs, changes in its phosphorylation have been detected by several groups. It is demonstrated that GluN2B phosphorylation at Tyr1472 site was involved in the enhanced adult seizure susceptibility after FSs (Chen et al., 2016). Furthermore, a selective long-term deficit in NMDA receptor-mediated ERK1/2 phosphorylation was observed in the hippocampus after FSs. There was a specific alteration in NR2A, but not NR2B in subunit tyrosine phosphorylation in adult (Chang et al., 2005). LTP in CA3-CA1 synapses was strongly reduced, which contributed to the insufficient activity of NMDARs. Whole cell recordings found a greater desensitization of NMDAR currents in teens after FSs, probably due to insufficient glycine site activation of NMDARs, as application of D-serine (a glycine site agonist) allowed LTP to return to control values of rats (Postnikova et al., 2021). Taken together, intervening on specific phosphorylation sites of NMDARs and downstream signals rather than directly regulating their expression may be more helpful to prevent long-term pathological changes after FSs. The dentate gyrus of rats showed impaired paired pulse suppression and excitation ratio, and increased VGLUT-1 (Vesicular Glutamate Transporter 1) immunoreactivity 10–12 weeks after FSs (Kwak et al., 2008)). Due to the importance of VGLUTs in maintaining low extracellular glutamate concentrations and their association with epilepsy syndromes (van der Hel et al., 2009; Santolini et al., 2017), it is highly recommended to conduct more research to discover more drug targets.

### 4.4 Genetics

Patients with *SCN1A* mutations often experience prolonged early-life FSs, raising the possibility that these events may influence epileptogenesis and lead to more severe adult phenotypes. To test this hypothesis, Dutton et al. subjected 21–23-day-old mice expressing the human *SCN1A* GEFS + mutation R1648H to prolonged hyperthermia, and then examined seizure and behavioral phenotypes during adulthood. They found that early-life FSs resulted in lower latencies to induced seizures, increased severity of spontaneous seizures, hyperactivity, and impairments in social behavior and recognition memory during adulthood (Dutton et al., 2017). The underlying sodium current of this mutant exhibited a significantly shifted hyperpolarizing inactivation threshold at room temperature and elevated temperature (Roemmich et al., 2021). Since this change is constitutive, it is likely to interact with thermally induced changes in other cellular properties, leading to sustained depolarization and thermally induced increases in seizure activity. Meta-analysis also revealed a genome-wide significant association for TLE and early-life FSs at the sodium channel gene cluster on chromosome 2q24.3 within an intron of the *SCN1A* gene (Kasperaviciute et al., 2013). Functional studies of the mutations showed that it caused biophysical defects of Na (V)1.2 and impaired its cell surface expression (Shi et al., 2012; Orlando et al., 2022). But more direct evidence for the therapeutic effect of intervention of *SCN1A* on adult diseases after FSs need to be provided. Massive parallel sequencing of *GABRB3* was performed in 416 patients with a range of epileptic encephalopathies and childhood-onset epilepsies. The results indicated that *GABRB3* mutations, which will reduce GABAergic receptors functions, are associated with a broad phenotypic spectrum of epilepsies and that reduced receptor function causing GABAergic disinhibition represents the relevant disease mechanism (Moller et al., 2017). Considering the important role of the GABAergic system in the neurological abnormalities induced by FSs, this mutation should receive more attention. In addition, adhesion G protein-coupled receptor V1 (ADGRV1) is potentially associated with FS-related epilepsy as a susceptibility gene, encoding a very large G protein-coupled receptor-1 (VLGR1), which is localized at synaptic junctions and cooperatively regulates synaptic function (Togashi et al., 2002). The variation in *ADGRV1* results in a significant loss of major functional domains of the VLGR1 protein. *ADGRV1* variants associated with FSs/epilepsy respond well to antiepileptic drugs, implying a clinical significance (Zhou et al., 2022).

### 4.5 DNA methylation levels

A cross-sectional pilot study investigated whether global DNA methylation levels (5-mC and 5-hmC markers) and DNMT isoforms (DNMT1, DNMT3a1, and DNMT3a2) expression would be different in hippocampal and neocortical tissues between controls and TLE patients with or without a history of FSs. Compared with the control group, the overall level of DNA methylation and the expression of DNMT3a2 subtype in the hippocampus of all TLE groups were lower, while the decline in the TLE group with a history of FSs was greater. Interestingly, compared with the control group and other TLE groups, the expression of DNMT3a1 in the hippocampus of TLE patients with FSs history was significantly reduced (de Nijs et al., 2019). They did not investigate the epigenetic and functional changes following the methylation changes. In addition, tri-methylation of histone 3 at Lys9 and Lys27 was decreased in the FSs group, suggesting a potential mechanism for the long-term effects of FSs on energy metabolism via histone methylation (Heng et al., 2016).

As previously mentioned, FSs caused adult memory deficits and are transmitted to the next-generation, mostly through the mother. For both generations, DNMT 1 is upregulated, leading to transcriptional repression of the synaptic plasticity protein, coilin, but not memory inhibitory protein phosphatase 1. DNMT inhibitors prevented the high expression of DNMT1 and hypermethylation of curly genes, reversing transgenerational memory deficits (Dai et al., 2019). The above results suggest that the effects of DNA methylation levels not only persist long term but may also persist across generations.

### 4.6 Ion channel

In the hippocampus of rats that had FSs, the long-lasting enhancement of the widely expressed intrinsic membrane conductance Ih converts the potentiated synaptic inhibition to hyperexcitability in a frequency-dependent manner (Chen et al., 2001). Kamal et al. determined long-term changes in neuronal excitability of rat hippocampal CA1 pyramidal cells after FSs. They showed that FSs induced an increase in the hyperpolarization-activated current Ih and a reduction in the amplitude of the slow afterhyperpolarization following FSs, which is likely to contribute to the hyperexcitability of the hippocampus 3–5 weeks after FS (Kamal et al., 2006). These studies have not been able to identify an intervention molecular target, but Harris et al. found that inhibiting TRPV1 receptors during FSs prevented learning deficits in young adult female rats (Harris et al., 2022). Whether modulation of other ion channels can also alter the long-term neurological effects caused by FSs and whether the downstream series of signaling pathways resulting from these current alterations are the true culprits remain unclear.

## 5 Conclusion

With FSs as one of the highest incidences in early childhood, it is important to assess and prevent the adverse long-term neurodevelopmental effects that FSs causes. In this review, the long-term adverse effects of FSs on neurodevelopment and the underlying mechanisms are systematically reviewed and summarized, and some specific novel targets with therapeutic value are specifically proposed. Overall, because the onset of FSs is precisely at the stage of rapid infantile neural-glial development, a series of neural-glial changes are triggered after FSs. Some protein or molecular alterations are long-term, even until disease onset in adulthood. Some alterations, although transient in short term, cause long-term evolution of some downstream signaling pathways, which may ultimately lead to disease.

Long term follow-up of more patients after FSs may be necessary, in conjunction with experimental animal models, to elucidate the complex effects of FSs on neurodevelopment. Indeed, several important questions remain open. First, is the frequency and duration of FSs linked to the type and severity of illness occurring later? Second, are there common lesions or targets in different diseases caused by FSs? Third, is there optimal time window for neuropsychiatric disorders resulting from FSs? Is prophylactic treatment necessary? If these issues can be addressed, they will undoubtedly better guide clinical treatment and greatly benefit patients. Gene mutations will have long-term stable effects in patients, which could be important underlying mechanism to explain the occurrence of adult disease after FSs. For example, *SCN1A* GEFS + mutations cause FSs and genetic epilepsies. However, it remains unclear how most individual mutations in *SCN1A* lead to seizures. Recent studies have identified a significant depolarizing shift in action potential threshold in parvalbumin expressing inhibitory hippocampal CA1 interneurons but not in firing properties of excitatory pyramidal neurons, suggesting that mutations in the same gene on different cells may cause different consequences (Das et al., 2021). Thus, if single-cell sequencing developed in recent years is used in the future, it will help to identify more specific molecules and also to explain shared mechanisms of different diseases. A variety of intravital imaging techniques that have revolutionized rapidly also offer more possibilities for long-term observations in preclinical studies. A general genetic strategy for precise control of copy number of fluorescently labeled molecules in cells could help visualizing long-term single-molecule dynamics *in vivo* (Liu et al., 2018). At the same time, probes for long-term *in vivo* imaging without autofluorescence have also been developed due to the disadvantages that cannot be easily overcome by fluorescent proteins, such as long maturation time, low brightness, photobleaching, broad emission spectrum, and sample autofluorescence. Although it can still only be applied to transparent zebrafish (Cardoso Dos Santos et al., 2020), it is also a new idea for long-term observation. Label free atraumatic large-scale photoacoustic microscopy methods have also emerged that can enable long-term imaging of angiogenesis in an undisturbed environment (Rebling et al., 2021). The application of these new methods can facilitate long-term observation after FSs and the elucidation of mechanisms. More powerful tools may also improve the situation in which a hundred flowers bloom but the main line is unclear in the current mechanism research. Based on that, future research should be engaged in discovering biomarkers of disease after FSs and critical drug targets, accelerating the development of drugs with less side effects, and providing more effective therapeutic strategies.
