# Supplementary material for: The long-term neurodevelopmental outcomes of febrile seizures and underlying mechanisms
Source: Front Cell Dev Biol. 2023 May 25;11:1186050. doi: 10.3389/fcell.2023.1186050 (PMC10248510; doi:10.3389/fcell.2023.1186050)
Supplement: Supplementary file 1 [file Table1.docx]

**Table 1. Possible mechanism of FSs**

| **Hypothesis** | **Potential targets** |
| --- | --- |
| Genetics | *SCN1A, SCN1B, SCN2A, GABRG2, SRP9, ADGRV1, PTGER3, PTGER10, IL-2, IL-6, IL-10, BSN, ERC2, GABRG2, HERC1, STX1B, CELF4, IFI44L, CD46,*  *ANO3, TMEM16C* |
| Ion channel activity | KCC2, NKCC1, TRPC3, TRPV1 |
| Inflammation | IL-6, IL-8, IL-1Ra, cappase-1/IL-1β, HMGB1, TNF-α |
| Respiratory alkalosis | Vagal TRPV1 activation |
| Others | Iron deficiency,  Immunoreactive-arginine vasopressin and  immunoreactive-somatostatin |
