# Supplementary material for: The long-term neurodevelopmental outcomes of febrile seizures and underlying mechanisms
Source: Front Cell Dev Biol. 2023 May 25;11:1186050. doi: 10.3389/fcell.2023.1186050 (PMC10248510; doi:10.3389/fcell.2023.1186050)
Supplement: Supplementary file 2 [file Table2.DOCX]

**Table 2. Possible mechanism of FSs recurrence**

| **Hypothesis** | **Potential targets** |
| --- | --- |
| Genetics | *GABRG2* |
| Ion channel activity | TRPV1 |
| Hyponatremia | None |
| Hypozinc | None |
